# Neuroimmunometabolic alterations and severity of depressive symptoms in people with HIV: An exploratory diffusion-weighted MRS study

**DOI:** 10.1177/23982128251335792

**Published:** 2025-04-29

**Authors:** Arish Mudra Rakshasa-Loots, Goabaone Diteko, Nicholas G. Dowell, Itamar Ronen, Jaime H. Vera

**Affiliations:** 1 Department of Global Health and Infection, Brighton and Sussex Medical School, University of Sussex, Brighton, UK; 2 Edinburgh Neuroscience, School of Biomedical Sciences, The University of Edinburgh, Edinburgh, UK; 3 University Hospitals Sussex NHS Foundation Trust, Brighton, UK; 4 Clinical Imaging Sciences Centre (CISC), Brighton and Sussex Medical School, University of Sussex, Brighton, UK

**Keywords:** Immunopsychiatry, neuroimaging, neuroinflammation, magnetic resonance spectroscopy, major depressive disorder

## Abstract

Depression is associated with inflammation in the periphery and the central nervous system. People with HIV are at greater risk for depression, which may in part be driven by sustained neuroinflammation, although individuals with severe depression are often excluded from studies of HIV-related co-morbidities. In this exploratory study, we aimed to explore the neuroimaging signatures of severe and persistent depression among people with HIV. We enrolled N = 20 adults with HIV in Brighton, UK, of whom n = 11 had a Patient Health Questionnaire-9 (PHQ-9) score ⩾15 and a history of receiving antidepressant medication. We used diffusion-weighted magnetic resonance spectroscopy (DW-MRS), an emerging neuroimaging technique sensitive to neuroinflammation, to assess neurometabolite diffusion in the anterior cingulate cortex. Participants also underwent standard magnetic resonance spectroscopy (MRS) to assess neurometabolite concentrations in the anterior cingulate cortex, and dynamic contrast-enhanced magnetic resonance imaging (DCE-MRI) to assess blood–brain barrier permeability in the whole brain and the thalamus. We observed a significant positive correlation between intracellular diffusion of creatine and depressive symptom severity (ρ = 0.46, p = 0.047). Increased creatine diffusion has previously been reported in conditions characterised by hypermetabolism and neuroinflammation, suggesting that worse depressive symptom severity in people with HIV may be correlated with neuroimmunometabolic alterations. Metabolite concentrations and blood–brain barrier permeability largely did not correlate with depressive symptom severity in this sample. In summary, we explored neuroimaging signatures of severe depression in people with HIV, including by applying diffusion-weighted magnetic resonance spectroscopy in this population. We report early evidence that worse depressive symptom severity in people with HIV may be correlated with neuroimmunometabolic dysfunction, evidenced by increased diffusion of creatine, likely reflecting hypermetabolism and neuroinflammation. Future research may aim to replicate these findings in larger and more diverse samples and compare the diffusion of neurometabolites between people with and without HIV living with severe depression.

## Introduction

Depression results in the greatest global disease burden among neuropsychiatric conditions (GBD 2019 Mental Disorders Collaborators, 2022). People with depression present with varied symptom profiles and, in up to 55% of these individuals, symptoms persist despite receiving first-line antidepressants (McIntyre et al., 2023). Given this heterogeneity, we now understand depression to comprise a constellation of distinct subtypes, rather than a monolithic disease. Identifying and targeting the relevant neurobiological mechanisms may enable effective interventions for each subtype of depression.

Inflammation is increasingly being recognised as a mechanism underlying a subtype of depression. Early-life increases in biomarkers of inflammation can predict the total number of depressive episodes later in life (Edmondson-Stait et al., 2022), and people who exhibit inflammation are more likely to be resistant to standard antidepressant treatments (Strawbridge et al., 2015). Conversely, anti-inflammatory medications can alleviate depressive symptoms (Cai et al., 2020; Nettis et al., 2021). Together, this evidence suggests that inflammation may drive a subtype of depression, termed ‘inflammatory depression’.

People with HIV are at three times greater risk for depression than people without HIV (Do et al., 2014). People with HIV also exhibit peripheral and central nervous system inflammation, evidenced by increased cytokine release in blood and microglial activation in the brain, which persists despite antiretroviral therapy (ART) (Babu et al., 2019; Vera et al., 2016). In addition to neuroinflammation, people with HIV also show increased blood–brain barrier (BBB) permeability (Atluri et al., 2015), which is in turn implicated in the pathophysiology of depression in the general population (Wu et al., 2022). Disruption of the BBB (via enlargement of the choroid plexus) has been observed to overlap with increases in neuroinflammation (indicated by greater translocator protein (TSPO) binding) in people with depression (Althubaity et al., 2022). It is thus possible that the increased risk for depression among people with HIV is linked, at least in part, to increased neuroinflammation and BBB permeability. However, people with HIV living with severe depression are often excluded from studies of HIV-related co-morbidities, resulting in limited understanding of whether neuroinflammation or BBB dysfunction may contribute to the severity of depression in this community.

Measuring neuroinflammation directly in humans remains challenging. One indirect in vivo measure of neuroinflammation is proton magnetic resonance spectroscopy (^1^H-MRS or simply MRS), which is used to measure concentrations of neurometabolites in localised brain regions non-invasively. Of relevance to neuroinflammation, the metabolites choline (Cho) and myo-inositol (mI) – widely considered to be markers of cell membrane turnover and glial cell activation, respectively – can be quantified using MRS (Quarantelli, 2015).

However, there are several challenges in using Cho or mI concentrations as biomarkers of neuroinflammation: elevations in Cho and mI may be confounded by variables such as participant age or alcohol consumption (Chang et al., 2013; Zahr et al., 2014) and changes in Cho concentrations may reflect processes such as (de)myelination in addition to neuroinflammation (Mader et al., 2008). Therefore, more sensitive and specific non-invasive measures of neuroinflammation are needed to precisely characterise the relationship between neuroinflammation and depression.

Diffusion-weighted magnetic resonance spectroscopy (DW-MRS) is an emerging non-invasive neuroimaging technique that can overcome some of the limitations of standard MRS. Whereas standard MRS quantifies concentrations of neurometabolites, DW-MRS estimates the intracellular diffusion rates (quantified as Apparent Diffusion Coefficients, ADCs) of neurometabolites in localised brain regions (Genovese et al., 2021). By combining the microstructural sensitivity of diffusion-weighted MR imaging (DW-magnetic resonance imaging (MRI)) with the compartmentalisation of neurometabolites, DW-MRS can offer higher specificity than DW-MRI to processes that selectively affect specific cell populations, such as neuroinflammation (microglia and astrocytes) and neurodegeneration (neurons) (Ronen and Valette, 2007). DW-MRS was recently shown to be sensitive to experimentally induced neuroinflammation in humans and may thus represent a useful non-invasive tool to explore the relationship between neuroinflammation and depression (De Marco et al., 2022). Measuring neuroinflammation and determining its influences on long-term clinical outcomes is of critical importance in people with HIV. DW-MRS may therefore be a promising technique for research involving people with HIV but has never before been applied in this context.

In this exploratory study, we aimed to explore the neuroimaging signatures of severe and persistent depression among people with HIV. Our primary objective was to determine the relationship between neuroinflammation and the severity of depressive symptoms in people with HIV. To achieve this objective, we used three neuroimaging techniques, each of which reflects a different aspect of the possible effects of neuroinflammation. Our secondary objective was to explore the relationships between these neuroimaging parameters and proteins in blood serum indicative of inflammation and neurological dysfunction.

## Methods

### Participants

For this cross-sectional exploratory study, we recruited adults with HIV receiving their regular clinical care at the Lawson Unit (Royal Sussex County Hospital) in Brighton, UK. All participants provided written informed consent prior to enrolment in the study. This study received ethical approval from the London–Surrey Borders Research Ethics Committee (reference 22/LO/0546).

Principal inclusion criteria for this study were participants aged 18 years or older with a documented HIV-1 infection, currently receiving a stable antiretroviral regimen and virally suppressed (plasma HIV RNA <50 copies/mL documented at screening). Participants were recruited into one of two study groups based on their nine-item Patient Health Questionnaire (PHQ-9) score at screening: high depressive symptom severity (‘HD’) or low depressive symptom severity (‘LD’). Participants were eligible for the HD group if they had a PHQ-9 score ⩾15 and a history of receiving antidepressant medication or eligible for the LD group if they had a PHQ-9 score ⩽7.

Principal exclusion criteria for this study included lack of capacity to provide informed consent, active hepatitis C or B infection on most recent documented laboratory tests, or a PHQ-9 score at screening outside the criteria of the LD or HD groups (i.e. 7 < PHQ-9 < 15). Participants were also excluded if they had impaired renal function (estimated glomerular filtration rate [eGFR] < 60 mL/min at screening), active neurological or neurodegenerative diseases, or non-affective psychiatric disorders unrelated to depression (schizophrenia, obsessive compulsive disorder, trauma- or stress-related disorders). Finally, participants with any contraindications to MRI (e.g. implanted medical devices or claustrophobia) were also excluded from this study.

### Questionnaires

At a screening visit, all participants completed the PHQ-9 as a measure of current depressive symptom severity. The PHQ-9 is a brief and well-validated screening tool which tests for key features of depression, including anhedonia, fatigue and suicidality (Kroenke et al., 2001). Although the PHQ-9 is not a diagnostic tool, a PHQ-9 score ⩾10 offers high sensitivity and specificity for the detection of major depressive disorder (Negeri et al., 2021). All participants also completed a sociodemographic and lifestyle questionnaire, designed to provide inclusive options for self-identification.

### Neuroimaging

**Figure 1.**
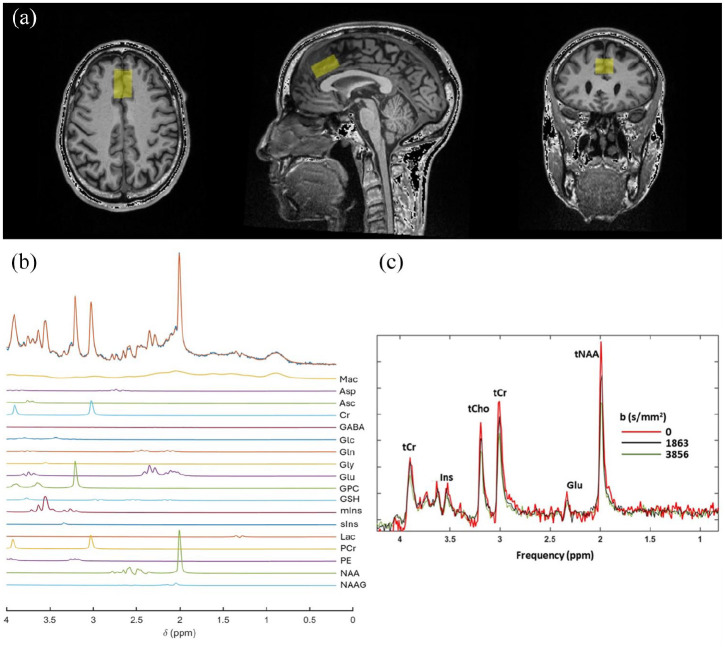
(a) Position of the volume of interest (VOI, in yellow) in the anterior cingulate cortex, (b) representative MRS spectrum from this VOI; upper row shows the spectrum and the LCModel fit, and the rows below show the fitted spectra of the individual metabolites included in the basis set and (c) representative DW-MRS spectra from this VOI at three b-values. tNAA: total NAA; Glu: glutamate; tCr: total creatine; tCho: total choline; Ins: myo-inositol.

All participants underwent neuroimaging within 28 days of screening, on a 3T Siemens Prisma MR scanner (Erlangen, DE) and 64 channel array receiver coil. Following a scout image and a T1-weighted anatomical image, a 2-cm (right-left, RL) × 1.5 cm (foot-head, FH) × 3 cm (anterior-posterior, AP) volume of interest (VOI) was positioned on the anterior cingulate cortex (ACC) (Figure 1).

MRS and DW-MRS were used to assess neurometabolite concentrations and diffusivity. We focused on the ACC, as inflammation in this region has previously been associated with depression in the general population (Holmes et al., 2018; Steiner et al., 2011). MRS data was acquired using a semi-LASER sequence (TE/TR = 30/3000 ms, 64 averages, GOIA-WURST pulses, acquisition bandwidth = 2500 Hz, number of complex time domain points = 2048). DW-MRS data were acquired with the same sequence (acquisition bandwidth = 2500 Hz, number of complex time domain points = 2048), equipped with a bipolar diffusion weighting scheme (Genovese et al., 2021) with three mutually orthogonal DW directions and three b-values (0, 1863 and 3856 s/mm^2^), 32 averages per DW condition and TE/TR = 80 ms/three heartbeats (cardiac synchronisation with pulse oximeter unit). A subsequent DW-MRS data set was acquired with the same acquisition parameters, with four averages and without water suppression, and the resulting water data were used for eddy current correction of the DW-MRS spectra. For both MRS and DW-MRS acquisitions, B0 shimming was performed using a three-stage FASTESTMAP, and water suppression was performed using VAPOUR.

Post-processing of MRS data was performed using an in-house MATLAB code and included spectral registration (Near et al., 2015) and eddy current correction based on the non-water suppressed data. Quantification was performed using LCModel (Provencher, 1993) using an appropriate basis set that included 18 metabolites and an experimentally acquired macromolecular baseline provided to us by Dr. Deelchand from University of Minnesota. Metabolites included in the basis set were: Aspartate (Asp), Ascorbate (Asc), Creatine (Cr), Phosphocreatine (PCr), GABA, Glucose (Glc), Glutamine (Gln), Glycine (Gly), Glutamate (Glu), Glycerophosphocholine (GPC), Myo-inositol (Ins), Scillo-inositol (sIns), Lactate (Lac), Phosphocholine (PCho), phosphorylethanolamine (PE). N-acetyl aspartate (NAA), N-acetyl aspartyl glutamate (NAAG), Taurine (Tau) and a macromolecular baseline (MM). Co-quantified metabolites: Cr + PCr, NAA + NAAG, GPC +PCho and Ins + Gly.

Scaling of the spectroscopic data to water was corrected for tissue-specific water T1, T2 and proton density values for grey matter (GM), white matter (WM) and cerebrospinal fluid (CSF) (Gasparovic et al., 2018). A Gannet tool (Edden et al., 2014) was used as following: tissue segmented maps were derived from the MPRAGE volume with SPM12, and subsequently applied VOI masks were used to obtain VOI-specific tissue fractions for GM, WM and CSF.

Post-processing of DW-MRS data included spectral registration (phase and frequency drift correction) with each diffusion-weighted condition and across directions within each b-value, eddy current correction and removal of spectra affected by motion (see, e.g., Genovese et al. (2021) for details). From the DW-MRS spectra, we quantified ADCs for three metabolites. From the MRS data, concentrations of nine metabolites were calculated relative to total creatine and concentrations of ten metabolites were calculated relative to water content within the VOI. Only metabolites for which Cramer Rao Lower Bounds (CLRB) was < 20% for all participants and all DW conditions were considered. CLRB values for all metabolites are available in Supplementary Materials.

To assess BBB permeability, whole-brain dynamic contrast-enhanced MRI (DCE-MRI) was acquired with a T_1_-weighted gradient echo pulse sequence: 100 volumes, time resolution 9.34 s, FA = 7 deg, voxel size = 2.5 mm^3^, total acquisition time = 15.5 min. The Gadolinium-based contrast agent Dotarem (gadoterate meglumine; 0.1 mMol/kg) was injected after 90 s of baseline scanning. Volume transfer rate constant (K_trans_, a proxy for BBB permeability) was extracted by fitting the Patlak model to the data. Segmentation of the mean baseline DCE images was performed with SPM to yield a whole-brain region(s) of interest (ROI), and FMRIB Software Tools (FSL) was used to extract mean whole-brain K_trans_ values from the maps. Thalamus ROI was taken from the Harvard-Oxford Subcortical Atlas and transformed into participant (native) space using Advanced Normalisation Tools (ANTs) and a diffeomorphic transformation. DCE-MRI data were evaluated in the thalamus (in addition to whole-brain) due to recent observations of BBB dysfunction and neuroinflammation in matched loci in the thalamus, and because of the hypothesised role of the thalamus in facilitating the passage of cytokines via blood vessels to trigger neuroinflammation (Tanaka et al., 2017; Van Vliet et al., 2020).

### Blood proteins

To quantify peripheral inflammatory and neurological biomarkers, we collected blood samples from all participants at the same study visit during which the PHQ-9 was administered. Blood samples were allowed to clot for 30 min at room temperature, and then centrifuged at 2000*g* for 10 min to extract serum. Serum samples were aliquoted and stored at −80°C within 2 h of collection at the Royal Sussex County Hospital Clinical Research Laboratory (Brighton, UK). Samples were subsequently shipped in a single batch on dry ice to the University of Edinburgh Biomolecular & Assay Core (Edinburgh, UK). Protein quantification was carried out in a single batch using Invitrogen™ ProcartaPlex Human Simplex and Combinable Panels (Thermo Fisher Scientific, UK) according to manufacturer’s instructions. Samples were diluted 1:500 for the CRP Simplex Panel and 1:100 for the YKL-40 Simplex Panel, with no sample dilutions for other analytes. Panels were analysed using a Luminex™ xMAP™ INTELLIFLEX DR-standard error (SE) System (Thermo Fisher Scientific, UK), and analyte concentrations in samples were obtained from standard curves using five-parameter logistic (5-PL) curves in the instrument software, BioPlex Manager.

All samples were assessed in triplicate by a single investigator, with values averaged across replicates for each analyte and sample. Only cases with coefficient of variation (%CV) < 20% across the three replicates were included. Where all replicates for a sample were below the detection range of the assay, a value that was half the minimum detection limit for that analyte was assumed, and where all replicates were above the detection range, a value twice the maximum detection limit for that analyte was assumed. Imputed values were assumed in this way for IL-17A (1 case), IFN-α (7), IL-1α (6), IL-1β (6), IL-8 (6) and MIP-1α (6), all below detection range and for CRP (1 case, above detection range), but for no other analytes.

### Statistical analysis

As this was an exploratory study, we did not carry out an a priori sample size estimation, and instead aimed to recruit a minimum feasible sample of 20 participants. Statistical analyses were performed in R v4.3.2. Group comparisons for participant characteristics were performed using Wilcoxon’s rank-sum tests (for continuous variables) or Pearson’s chi-square tests (for categorical variables). The majority of neuroimaging parameters were non-normally distributed (Supplementary Figure 1); thus, we used non-parametric statistical tests for analysis.

Partial Spearman’s correlations adjusted for fractional WM volumes were calculated for DW-MRS and MRS parameters with PHQ-9 score. Spearman’s correlations were calculated for values of DCE-MRI parameters with PHQ-9 score and for blood proteins with PHQ-9 score and all neuroimaging parameters. In sensitivity analyses, partial correlations were calculated for neuroimaging parameters with PHQ-9 score, to control for any relevant sociodemographic or clinical variables that differed significantly between participant groups. These adjustments were made for DW-MRS and MRS parameters in addition to controlling for fractional WM volumes. All p-values (except for comparisons of participant characteristics) were corrected for multiple comparisons using the False Discovery Rate (FDR) method to yield corrected q-values. Given that this was an exploratory study with a small sample, when interpreting our findings, we explored the magnitude of the correlations as well as their statistical significance to avoid an overemphasis on p-values (Boscardin et al., 2024). We used Funder and Ozer’s (2019) framework for interpreting the effect sizes, with correlations greater than 0.30 considered large effects, while noting that very large effects may likely be overestimated due to limited statistical power.

## Results

### Participant characteristics

**Figure 2.**
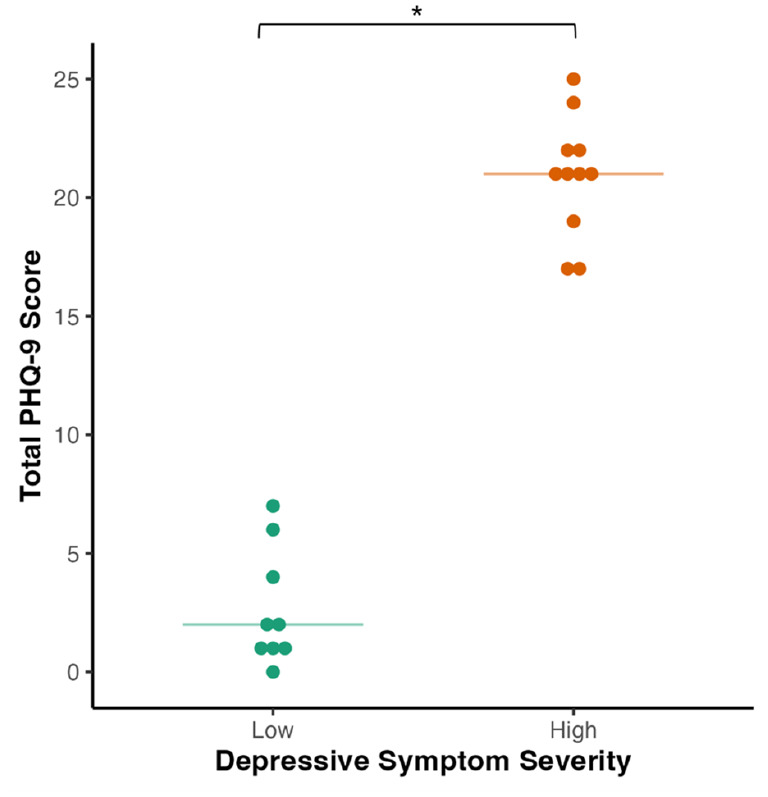
Distribution of total score on the nine-item Patient Health Questionnaire (PHQ-9) for high depressive symptom severity (HD) and low depressive symptom severity (LD) groups. Horizontal lines indicate group medians. * indicates p < 0.05.

Participants were N = 20 people with HIV, with median [interquartile range (IQR)] age of 50 [45, 54] years, of whom 90% self-identified as men and 80% as White (Table 1). Median [IQR] PHQ-9 score overall was 17.0 [2, 21]. Participants in the HD group (n = 11) reported significantly higher depressive symptom severity than those in the LD group (n = 9), with median [IQR] PHQ-9 score of 21 [20, 22] versus 2 [1, 4] (p < 0.001) (Figure 2). Both groups were comparable on most sociodemographic characteristics, except participants in the HD group reported fewer years of formal education (14 versus 19 years, p = 0.013). As anticipated by the study design, experience of treatment for depression differed between groups: a significantly greater proportion of participants in the HD group reported ever taking antidepressants (100% versus 44%, p = 0.008) and currently receiving psychotherapy (45% versus 0%, p = 0.038). Participants in the HD group also showed lower absolute CD4 count than those in the LD group (606 versus 855, p = 0.023). Participants were receiving a wide range of ART and antidepressant regimens, which did not differ significantly by group (Supplementary Table 1).

**Table 1.** Sociodemographic and clinical characteristics of participants overall and stratified by depressive symptom severity group.

Characteristic	Overall, N = 20	High depression severity, N = 11	Low depression severity, N = 9	p
Age (years), Median (IQR)	50.5 (45.0, 54.0)	51.0 (47.0, 54.0)	49.0 (45.0, 54.0)	0.8
Sex, n (%)				> 0.9
Female	1 (5.0%)	1 (9.1%)	0 (0%)	
Male	19 (95%)	10 (91%)	9 (100%)	
Gender, n (%)				> 0.9
Man	18 (90%)	9 (82%)	9 (100%)	
Man and Non-binary	1 (5.0%)	1 (9.1%)	0 (0%)	
Woman	1 (5.0%)	1 (9.1%)	0 (0%)	
Sexual Orientation, n (%)				0.6
Gay / lesbian / homosexual	17 (85%)	10 (91%)	7 (78%)	
Straight / heterosexual	3 (15%)	1 (9.1%)	2 (22%)	
Ethnicity, n (%)				0.5
Black–African	1 (5.0%)	0 (0%)	1 (11%)	
Latino / Latina / Latinx / Hispanic	1 (5.0%)	1 (9.1%)	0 (0%)	
Mixed race	1 (5.0%)	0 (0%)	1 (11%)	
White–Non-UK	3 (15%)	1 (9.1%)	2 (22%)	
White–UK	13 (65%)	8 (73%)	5 (56%)	
White – UK & Black–Caribbean	1 (5.0%)	1 (9.1%)	0 (0%)	
Years of Education, Median (IQR)	16.5 (13.5, 19.3)	14.0 (11.5, 16.0)	19.0 (17.0, 20.0)	**0.013**
Smoking, n (%)	1 (5.0%)	1 (9.1%)	0 (0%)	> 0.9
Alcohol Use, n (%)	13 (65%)	7 (64%)	6 (67%)	> 0.9
Recreational Drug Use, n (%)	2 (10%)	2 (18%)	0 (0%)	0.5
Currently Taking Antidepressants, n (%)	13 (65%)	9 (82%)	4 (44%)	0.2
Ever Taken Antidepressants, n (%)	15 (75%)	11 (100%)	4 (44%)	**0.008**
Currently Receiving Psychotherapy, n (%)	5 (25%)	5 (45%)	0 (0%)	**0.038**
Ever Received Psychotherapy, n (%)	12 (60%)	9 (82%)	3 (33%)	0.065
HIV RNA Viral Load < 40 copies/mL, n (%)	20 (100%)	11 (100%)	9 (100%)	
CD4 Count, Median (IQR)	686.5 (558.0, 804.0)	606.0 (515.0, 709.0)	855.0 (689.0, 966.0)	**0.023**

Participant groups were determined at screening using the Patient Health Questionnaire (PHQ-9) score as High Depression Severity (HD, PHQ-9 ⩾ 15 and history of using antidepressant medication) or Low Depression Severity (LD, PHQ-9 ⩽ 7). Participants could select multiple genders, sexual orientations or ethnicities. HIV RNA viral load and CD4 count were extracted from the most recent available clinical record for each participant.

*p* values < 0.05 are highlighted in bold text.

### Neurometabolite diffusion and depressive symptom severity

**Figure 3.**
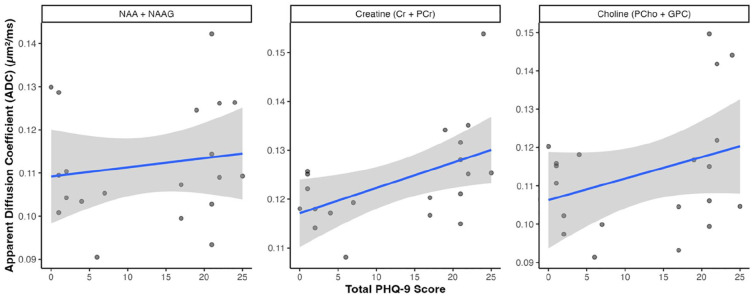
Neurometabolite diffusion and depressive symptom severity. Correlation between measures of neurometabolite diffusion (apparent diffusion coefficient, ADC) in the anterior cingulate cortex quantified using diffusion-weighted magnetic resonance spectroscopy and depressive symptom severity (total PHQ-9 score).

Summary statistics for all neuroimaging parameters are available in Supplementary Table 2. Diffusion of neurometabolites in the ACC was quantified as ADCs (Figure 3). ADC for creatine (corrected for fractional WM volume) was significantly and positively correlated with PHQ-9 score (ρ = 0.46, p = 0.047). However, this correlation did not survive FDR correction (q = 0.143). There was also a modest correlation of choline ADC (ρ = 0.21) with PHQ-9 score, but this was not statistically significant (q = 0.592). ADC for NAA was not correlated with PHQ-9 score (ρ = 0.04, q = 0.865).

As years of education and CD4 count differed significantly between the participant groups, we calculated partial Spearman’s correlations adjusted for these variables (separately) for DW-MRS parameters (Supplementary Table 3). Findings remained consistent after adjusting for CD4 cell counts. After adjusting for years of education, the correlation between ADC of creatine and PHQ-9 score was no longer significant (ρ = 0.25, p = 0.323, q = 0.945). ADC for creatine (corrected for fractional WM volume) was significantly correlated with years of education (ρ = –0.70, p < 0.001). All correlation coefficients and associated p-values are available in Supplementary Table 4.

### Neurometabolite concentrations and depressive symptom severity

**Figure 4.**
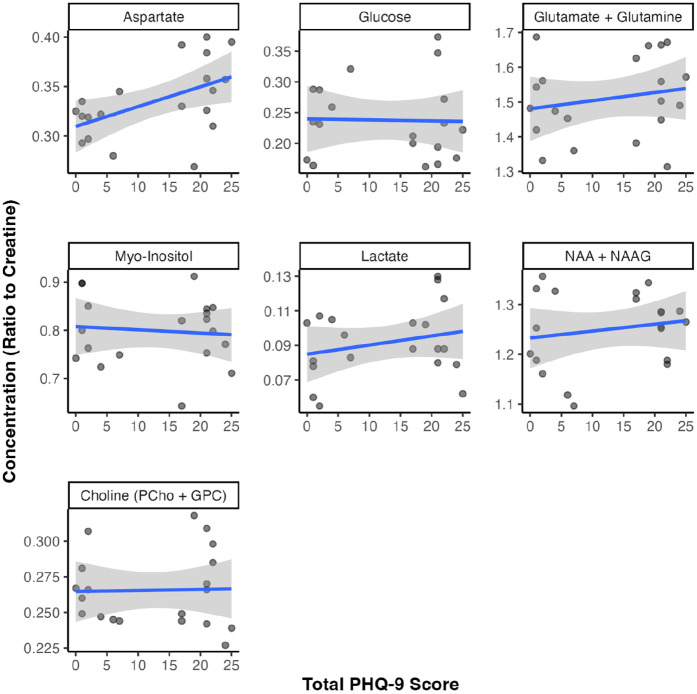
Neurometabolite concentrations and depressive symptom severity. Correlation between measures of neurometabolite concentrations (relative to total creatine) in the anterior cingulate cortex quantified using magnetic resonance spectroscopy and depressive symptom severity (total PHQ-9 score).

Concentrations of neurometabolites in the ACC were quantified relative to total creatine (Figure 4) and water content within the VOI (Supplementary Figure 2). After adjusting for fractional WM volume, creatine-referenced aspartate was significantly and moderately correlated with PHQ-9 score (ρ = 0.54, p = 0.016), though this correlation did not survive FDR correction (q = 0.115). Water-referenced aspartate also trended towards a correlation with PHQ-9 score (ρ = 0.43, p = 0.065, q = 0.527). None of the other metabolite concentrations, either referenced to creatine or water, were significantly associated with PHQ-9 score (all|ρ| < 0.30, q > 0.05). Findings remained largely consistent when adjusting for years of education or CD4 count (Supplementary Table 3).

### BBB permeability and depressive symptom severity

**Figure 5.**
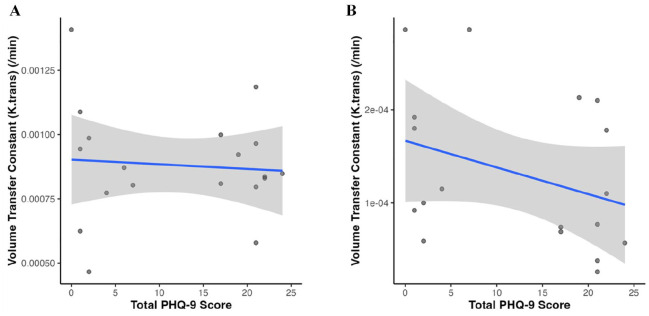
Blood–brain barrier permeability and depressive symptom severity. Correlation between measures of blood–brain barrier permeability (volume transfer rate constant, K_trans_) quantified using dynamic contrast-enhanced magnetic resonance imaging for the (A) whole brain (n = 19) and (B) thalamus (n = 18) and depressive symptom severity (total PHQ-9 score).

DCE-MRI data were not acquired for one participant who chose to withdraw from the scanning, and thalamus ROI was not available for one additional participant. Volume transfer rate constant (K_trans_) for the whole-brain ROI was not significantly correlated with PHQ-9 score (ρ = –0.15, q = 0.537). Similarly, K_trans_ for the thalamus ROI was not significantly correlated with PHQ-9 score, though the magnitude of this correlation was larger than that for the whole-brain ROI (ρ = –0.38, q = 0.257) (Figure 5). Findings remained consistent when adjusting these correlations for years of education or CD4 count (Supplementary Table 3).

### Neuroimaging parameters and blood proteins

We quantified 20 inflammatory and neurological proteins in blood serum samples, summary statistics for which are available in Supplementary Table 2. Of these 20 proteins, only NGF-β showed a significant correlation with PHQ-9 score (ρ = 0.48, p = 0.032), although this did not survive FDR correction (q = 0.640) (Supplementary Figure 3).

**Figure 6.**
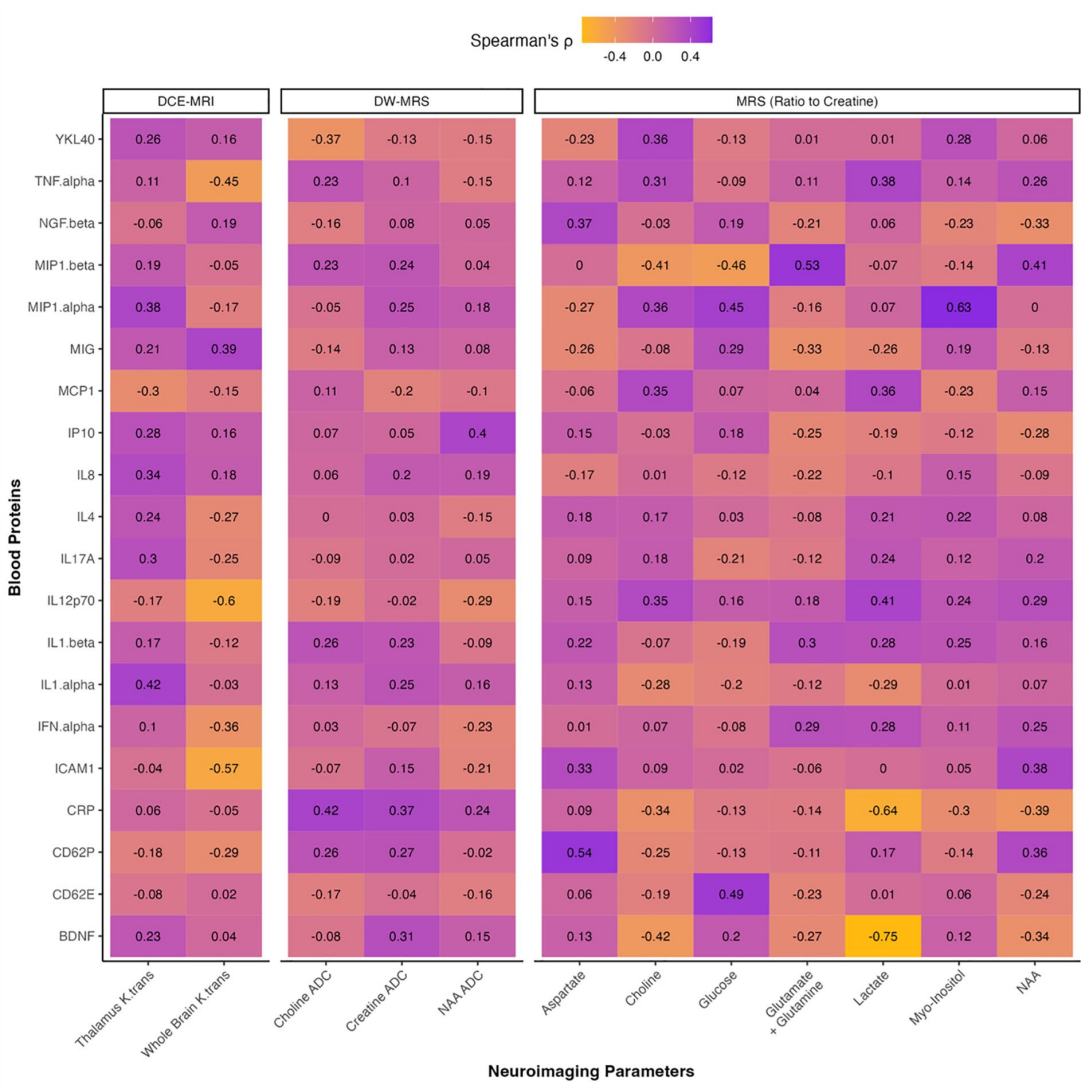
Neuroimaging parameters and blood proteins. Heatmap of Spearman’s correlations for neuroimaging parameters with concentrations of inflammatory and neurological proteins quantified in blood serum. Dynamic contrast-enhanced MRI (DCE-MRI) was used to assess blood–brain barrier permeability (volume transfer rate constant, K_trans_) in the thalamus and whole brain, diffusion-weighted MRS used to assess intracellular diffusion (apparent diffusion coefficient, ADC) of neurometabolites in the anterior cingulate cortex, and standard MRS used to assess concentrations (relative to creatine) of neurometabolites in the anterior cingulate cortex. Proteins in blood were quantified using Luminex™ immunoassays. Strength of correlations is represented by a colour gradient from −1.0 (bright yellow) to +1.0 (bright purple), with inlaid text in tiles indicating these magnitudes.

We then explored whether the neuroimaging parameters measured in this study may be correlated with these peripheral markers of inflammation and neurological dysfunction (Figure 6). While we focused on the magnitudes of these correlations in our interpretation, those correlations which met the threshold for statistical significance before FDR correction are highlighted in Supplementary Figure 4. ADCs of metabolites quantified using DW-MRS did not correlate strongly with any blood proteins (all|ρ| < 0.43, p > 0.075). Whole-brain K_trans_ was negatively correlated with IL-12p70 (ρ = –0.60, p = 0.009) and ICAM-1 (ρ = –0.57, p = 0.016). Creatine-referenced concentrations of several metabolites were also strongly correlated with peripheral markers, most notably lactate with BDNF (ρ = –0.75, p < 0.001) and CRP (ρ = –0.64, p = 0.008) and myo-inositol with MIP-1α (ρ = 0.63, p = 0.006). After FDR correction, these correlations were no longer statistically significant (all q > 0.05), although creatine-referenced lactate trended towards a significant correlation with BDNF (q = 0.050).

## Discussion

In this exploratory study, we applied diffusion-weighted MRS for the first time in a small sample of people with HIV. We aimed to explore the relationships of neuroimaging parameters with severe and persistent depression in people with HIV.

A key finding from this study is the moderate positive correlation of creatine ADC (measured using DW-MRS) with depressive symptom severity, indicating that participants with more severe depressive symptoms exhibited higher intracellular diffusion of creatine in the ACC. In recent DW-MRS studies, increased creatine ADC has been reported in patients with neuropsychiatric lupus (Ercan et al., 2016), a condition characterised by neuroinflammation, as well as in participants with ischaemic stroke (Genovese et al., 2022). In a DW-MRS study performed on a cohort of patients with multiple sclerosis, creatine ADC was found to be significantly lower in the thalamus and in normal appearing WM when compared to healthy controls (Bodini et al., 2018). This finding was interpreted as related to energy dysregulation in MS, either reflecting a pathological impairment of intercellular creatine transport or reduced phosphocreatine consumption secondary to impaired function of brain creatine kinase (CK-B). In addition, the increase in creatine ADC has been associated with the increase in metabolic activity in the visual cortex in a functional DW-MRS study (Branzoli et al., 2013). Creatine concentrations are shown to be higher in glial cells than in neurons, and animal models with SIV infection (the non-human primate analogue of HIV) revealed increased creatine concentrations concurrent with neuroinflammatory responses (Chang et al., 2013; Ratai et al., 2011). Given this previous evidence, the intracellular diffusion of creatine may be considered an indirect indicator for neuroimmunometabolic alterations. In this context, our preliminary findings suggest that severe and persistent depression in people with HIV may involve underlying hypermetabolism and neuroinflammation.

Notably, the correlation between creatine ADC and depressive symptom severity was attenuated when accounting for years of education, whereas creatine ADC was strongly and negatively correlated with years of education (ρ = –0.70). Years of education are a well-established proxy for socioeconomic status, higher levels of which can be protective against depression (Freeman et al., 2016). Conversely, lower socioeconomic status is associated with increased inflammation (Muscatell et al., 2020), poorer metabolic health (Stephens et al., 2020) and wider risk factors for depression, such as early life adversity or trauma (Yazawa et al., 2022). Our findings thus suggest that socioeconomic status may be correlated with creatine diffusion in the brain (possibly through cross-talk with inflammatory or metabolic pathways) and in turn influence depressive symptom severity.

We also explored the relationship of BBB permeability (measured using DCE-MRI) with depressive symptom severity. We did not find significant correlations between the volume transfer rate constant (K_trans_, a proxy BBB permeability) and depressive symptom severity. Increased BBB permeability has previously been reported in people with HIV, with strong evidence that HIV viral proteins may result in reduced integrity of the BBB (Chaganti et al., 2019; Strazza et al., 2011). In people with depression as well, increased BBB permeability has been observed, including in the thalamus (Shang et al., 2024). However, we did not find a significant correlation between BBB permeability and depressive symptom severity. Since all participants in our study had been with HIV for several years, long-term compromise of BBB integrity due to HIV may perhaps overshadow any subtler correlations between BBB permeability and depressive symptoms. Indeed, the median K_trans_ value for our participant sample (0.848 × 10^−3^ min^−1^) was higher than that recently reported in the group of participants with major depressive disorder (0.557 × 10^−3^ min^−1^) (Shang et al., 2024). Thus, the lack of correlation between BBB permeability and depressive symptom severity observed in our study may be partly driven by HIV-related BBB disruption.

We also observed nominally significant correlations between certain neurometabolites in the ACC and inflammatory or neurological proteins in blood serum. In particular, lactate concentration in the ACC was strongly correlated with peripheral BDNF concentration. The increase in brain lactate concentrations has been reported in depression as well as other psychiatric conditions such as bipolar disorder and schizophrenia (Chu et al., 2013; Ernst et al., 2017; Rowland et al., 2016). We extend these findings and report a possible link between increased brain lactate concentrations, indicative of hypermetabolism, and decreased peripheral BDNF concentrations, a well-established marker for depression. We also found that myo-inositol concentration in the ACC was strongly and positively correlated with peripheral MIP-1α, a chemokine involved in the inflammatory response. This finding aligns with previous studies which have reported associations between brain myo-inositol concentrations and peripheral chemokine concentrations in people with HIV and suggest an overlap between astrocytic activation in the brain and the peripheral inflammatory response (Vera et al., 2012).

As this was an exploratory study, we had a small sample size (N = 20), which is a major caveat to these results. Small sample sizes result in issues such as reduced statistical power to detect true underlying effects, possible underestimation or inflation of true effect sizes and risk of overfitting (Button et al., 2013; Yarkoni and Westfall, 2017). Therefore, our findings must be interpreted with caution. We have attempted to mitigate some of these issues by using simple correlation analyses rather than more complex models, limiting the number of covariates to one (or two, in the case of standard MRS data) to minimise the risk of overfitting, and reporting FDR-corrected q-values to account for multiple comparisons. Importantly, the correlations reported in this study did not survive FDR correction, but the magnitudes of these correlations suggest that at least some of these effects may be indicative of true underlying relationships. Very large effect sizes – especially in exploratory research linking biological and behavioural variables – are unlikely to be fully replicable in larger samples (Funder and Ozer, 2019). Nevertheless, as DW-MRS has never been applied in the context of HIV before, we argue that our findings (when interpreted with appropriate caveats) offer useful directions for future research, despite the small sample size of the current study.

Additional limitations for this study include its cross-sectional design, which limits any inferences about the causality or temporality of relationships between neurometabolite diffusion and depressive symptom severity. Due in part to the small sample size of our study, we used correlation analyses rather than regression modelling. Future studies with sufficient statistical power may seek to carry out linear (if recruiting participants throughout the depressive symptom severity spectrum) or quantile (if recruiting participants with high and low depressive symptom severity) regression with adjustments for relevant covariates to further explore these relationships. Our participant sample was also primarily white (80%), male (90%) and older (median age 50 years). This sample is broadly representative of the population of people with HIV in Brighton, UK. Nevertheless, these findings may not be immediately generalisable to other population groups, including women with HIV and people with HIV in the Global South.

Despite these limitations, this study makes a novel contribution to the literature by exploring neuroimaging signatures of severe depression in people with HIV, including by applying DW-MRS for the first time in this population. We offer early evidence that worse depressive symptom severity in people with HIV may be correlated with neuroimmunometabolic dysfunction, evidenced by increased diffusion of creatine, likely reflecting hypermetabolism and neuroinflammation. Future research may attempt to overcome some of the limitations of our exploratory study, by replicating these findings in larger and more diverse participant samples and assessing changes in DW-MRS parameters alongside changes in depressive symptom severity longitudinally. Regional differences exist in alterations in metabolite concentrations in the context of depression (Xie et al., 2023) or HIV (Salan et al., 2023), and although investigations of metabolite diffusion in these contexts are sparse, it is plausible that this diffusion may also vary by brain region. While this exploratory study focused on the ACC, future studies should thus investigate these imaging parameters in other brain regions implicated in depression, such as the prefrontal cortex or putamen. Given the changes in DW-MRS signal previously reported in response to lipopolysaccharide (LPS)-induced inflammation, future research may also quantify LPS in blood samples of people with HIV and severe depression to explore possible correlations with inflammatory markers and depressive symptom severity (Birg et al., 2024; De Marco et al., 2022). It will also be useful to compare neurometabolite diffusion between participants with and without HIV who are experiencing severe depression, to determine whether the DW-MRS signatures of severe depression observed in this study may be specific to people with HIV. In summary, intracellular diffusion of neurometabolites as measured using DW-MRS may be useful, non-invasive, and novel parameters to explore the neurobiological mechanisms underlying depression, including in people with HIV.

## Supplemental Material

sj-docx-1-bna-10.1177_23982128251335792 – Supplemental material for Neuroimmunometabolic alterations and severity of depressive symptoms in people with HIV: An exploratory diffusion-weighted MRS study Supplemental material, sj-docx-1-bna-10.1177_23982128251335792 for Neuroimmunometabolic alterations and severity of depressive symptoms in people with HIV: An exploratory diffusion-weighted MRS study by Arish Mudra Rakshasa-Loots, Goabaone Diteko, Nicholas G. Dowell, Itamar Ronen and Jaime H. Vera in Brain and Neuroscience Advances
