# Supplementary material for: Neuroimmunometabolic alterations and severity of depressive symptoms in people with HIV: An exploratory diffusion-weighted MRS study
Source: Brain Neurosci Adv. 2025 Apr 29;9:23982128251335792. doi: 10.1177/23982128251335792 (PMC12041680; doi:10.1177/23982128251335792)
Supplement: sj-docx-1-bna-10.1177_23982128251335792 – Supplemental material for Neuroimmunometabolic alterations and severity of depressive symptoms in people with HIV: An exploratory diffusion-weighted MRS study [file sj-docx-1-bna-10.1177_23982128251335792.docx]

**SUPPLEMENTARY MATERIALS**

**Title of article:**
Neuroimmunometabolic alterations and severity of depressive symptoms in people with HIV: an exploratory diffusion-weighted MRS study

**Authors**:
Arish Mudra Rakshasa-Loots, Goabaone Diteko, Nicholas G. Dowell, Itamar Ronen, Jaime H. Vera

**Correspondence**:
Arish Mudra Rakshasa-Loots ([Arish.MRL@ed.ac.uk](mailto:Arish.MRL@ed.ac.uk))

***Table of Contents***

| Supp Fig 1: Distribution of neuroimaging outcome variables. | p.1 |
| --- | --- |
| Supp Fig 2: Neurometabolite concentrations (relative to water) and depressive symptom severity. | p.2 |
| Supp Fig 3: Blood proteins and depressive symptom severity. | p.3 |
| Supp Fig 4: Neuroimaging and blood biomarkers, significant correlations only. | p.4 |
| Supp Table 1: Antiretroviral and antidepressant treatment regimens for all participants. | p.5 |
| Supp Table 2: Summary statistics for all neuroimaging parameters and blood proteins. | p.6 |
| Supp Table 3: Spearman’s correlations for all neuroimaging parameters and depressive symptom severity, adjusted for years of education or CD4 count. | p.7 |
| Supp Table 4: Spearman’s correlations for all neuroimaging parameters and blood proteins with depressive symptom severity. | p.9 |
| Supp Table 5: Cramer-Rao Lower Bounds for MRS data. | p.11 |
| Supp Table 6: Cramer-Rao Lower Bounds for DW-MRS data. | p.12 |


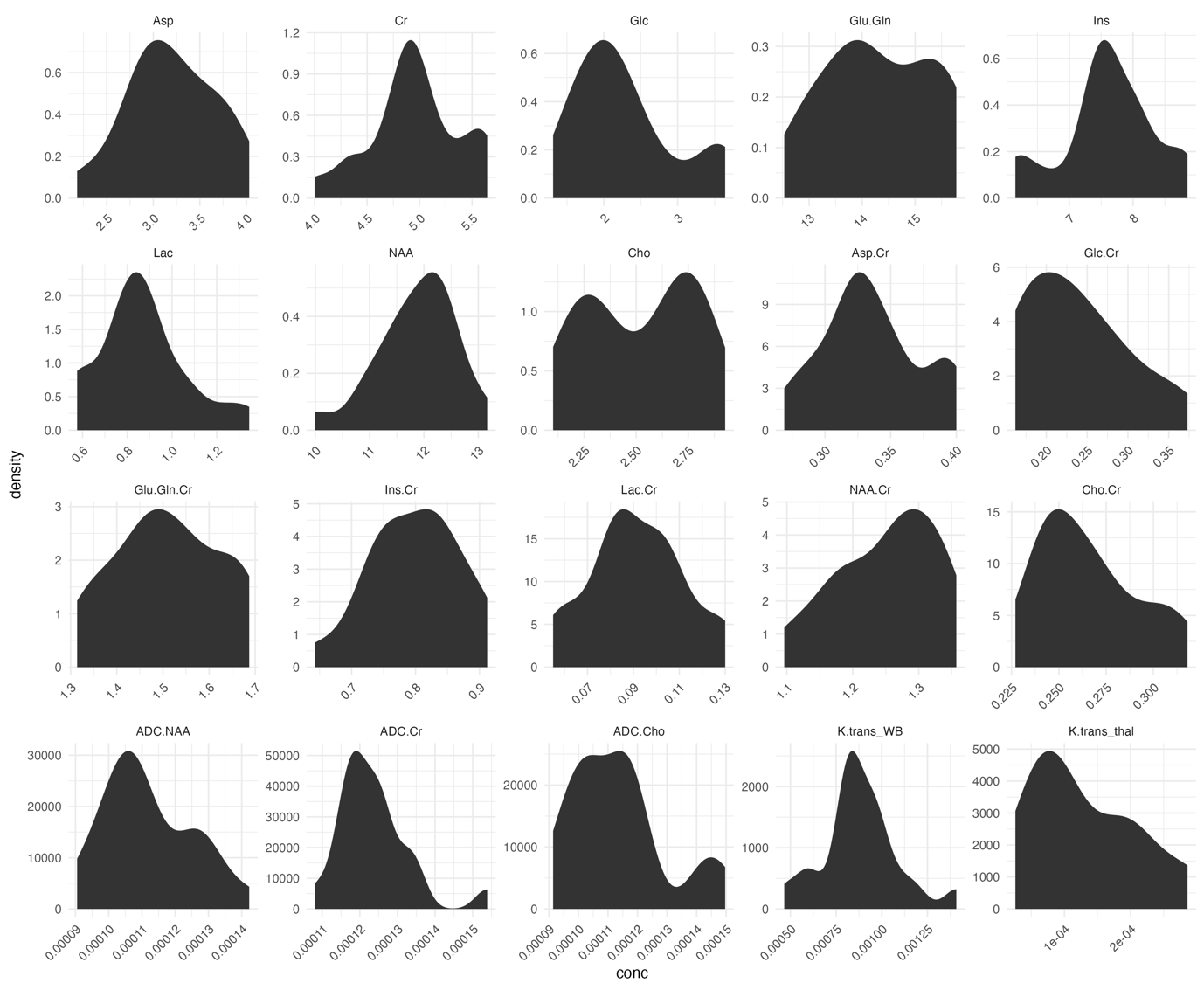


***Supp Figure 1*** *| Distribution of neuroimaging outcome variables.*


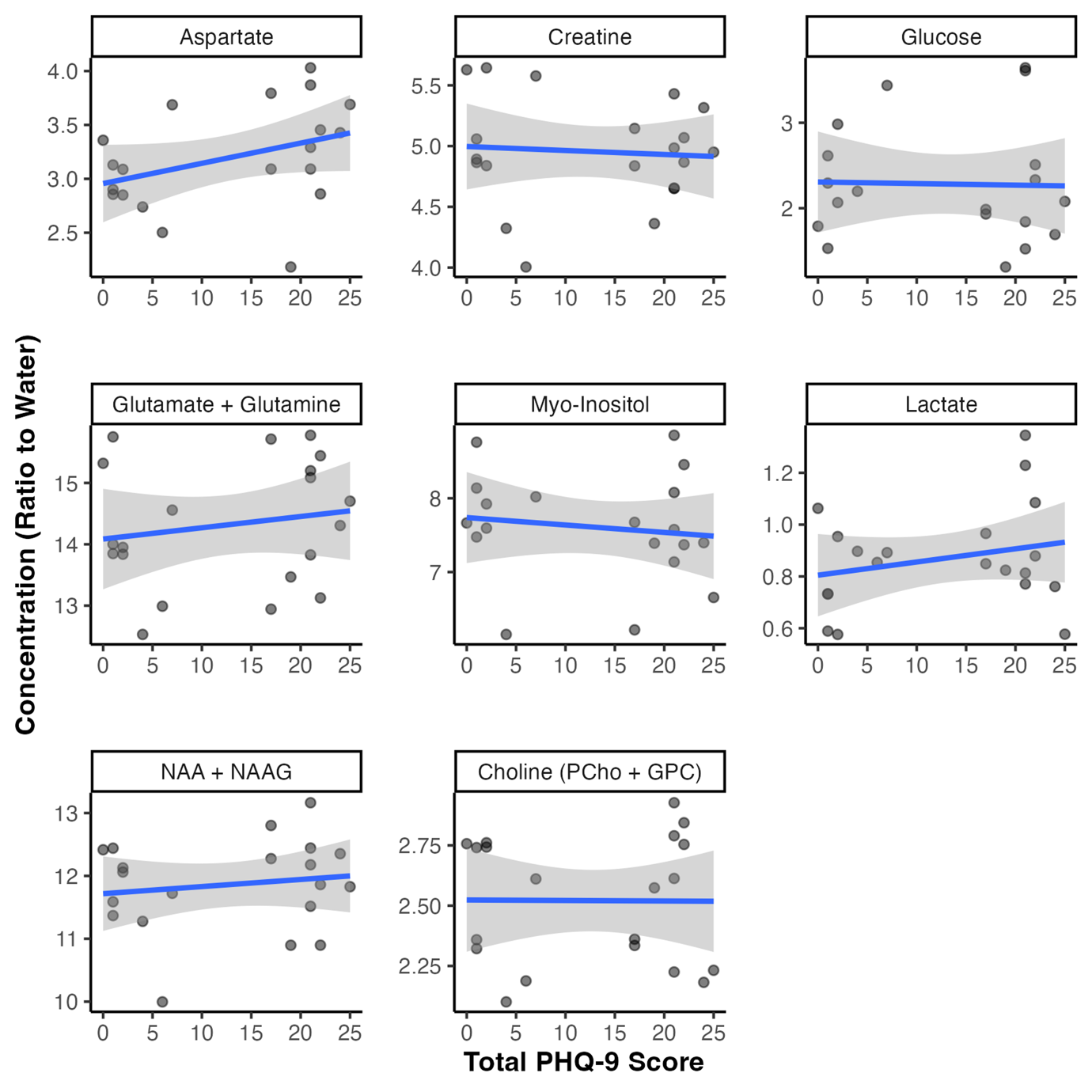


***Supp Figure 2*** *|* ***Neurometabolite concentrations (relative to water) and depressive symptom severity.*** *Correlation between measures of water-referenced neurometabolite concentrations in the anterior cingulate cortex quantified using magnetic resonance spectroscopy and depressive symptom severity (total PHQ-9 score).*


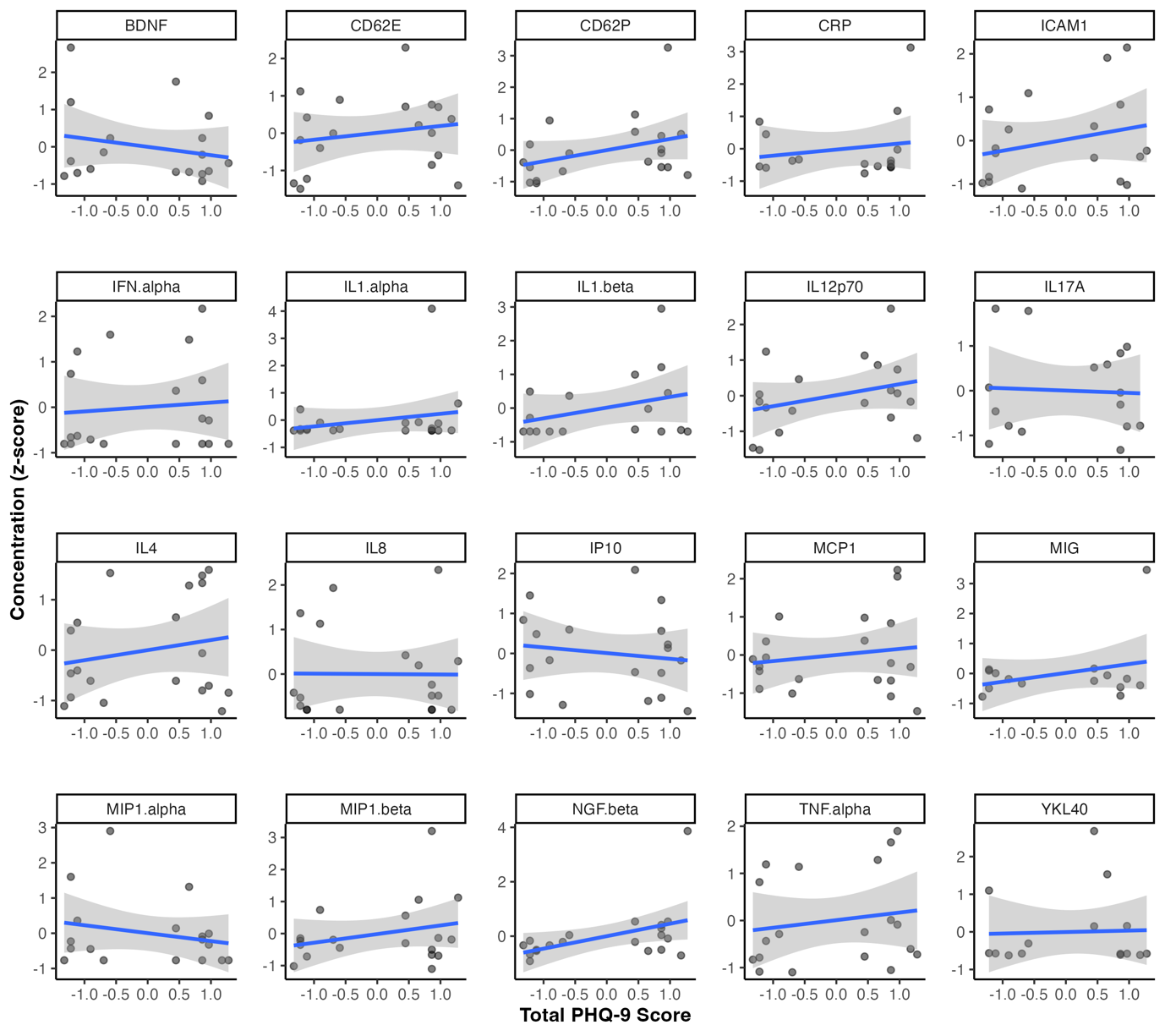


***Supp Figure 3*** *|* ***Blood protein concentrations and depressive symptom severity.*** *Correlations between concentrations of inflammatory and neurological proteins in blood serum (transformed to z-scores) quantified using Luminex™ immunoassays and depressive symptom severity (total PHQ-9 score).*


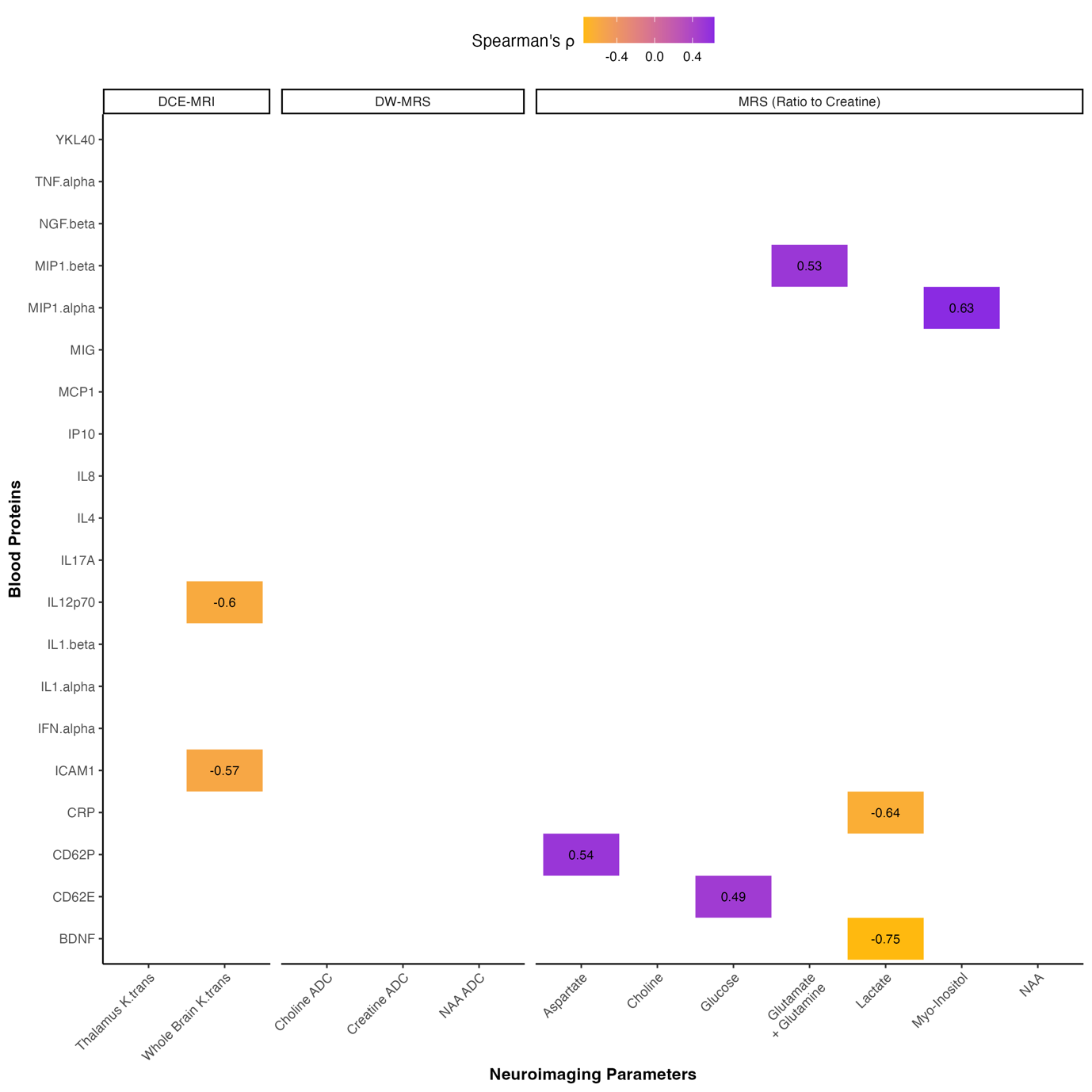


***Supp Figure 4*** *|* ***Neuroimaging and blood biomarkers, significant correlations only.*** *Spearman’s correlations between concentrations of inflammatory and neurological proteins in blood serum and neuroimaging parameters. Only correlations with p < 0.05 are shown. In-laid text in tiles shows the magnitude of each correlation. Dynamic contrast-enhanced MRI (DCE-MRI) was used to assess blood-brain barrier permeability (volume leakage constant, K_trans_) in the thalamus and whole brain, diffusion-weighted MRS used to assess intracellular diffusion (apparent diffusion coefficient, ADC) of neurometabolites in the anterior cingulate cortex, and standard MRS used to assess concentrations (relative to creatine) of neurometabolites in the anterior cingulate cortex. Proteins in blood were quantified using Luminex™ immunoassays.*

***Supp Table 1 |*** *Antiretroviral and antidepressant treatment regimens for all participants.*

| **Characteristic** | **Overall**, N = 20 | **High Depression Severity**, N = 11 | **Low Depression Severity**, N = 9 | **p-value** |
| --- | --- | --- | --- | --- |
| **ART Regimen, n (%)** | | | | 0.9 |
| Delstrigo | 7 (35%) | 4 (36%) | 3 (33%) |  |
| Biktarvy | 2 (10%) | 1 (9.1%) | 1 (11%) |  |
| Cabotegravir + Rilpivirine LAI | 2 (10%) | 1 (9.1%) | 1 (11%) |  |
| Odefsey | 2 (10%) | 2 (18%) | 0 (0%) |  |
| Descovy | 1 (5.0%) | 1 (9.1%) | 0 (0%) |  |
| Darunavir/ Emtricitabine/ Tenofovir/ Ritonavir | 1 (5.0%) | 1 (9.1%) | 0 (0%) |  |
| Dovato | 1 (5.0%) | 0 (0%) | 1 (11%) |  |
| Genvoya | 1 (5.0%) | 0 (0%) | 1 (11%) |  |
| Nevirapine/ Emtricitabine/ Tenofovir | 1 (5.0%) | 1 (9.1%) | 0 (0%) |  |
| Rilpivirine/Emtricitabine+tenofovir | 1 (5.0%) | 0 (0%) | 1 (11%) |  |
| Triumeg | 1 (5.0%) | 0 (0%) | 1 (11%) |  |
| **Antidepressant Regimen, n (%)** | | | | 0.5 |
| None | 7 (35%) | 2 (18%) | 5 (55%) |  |
| Mirtazipine | 3 (15%) | 1 (9.1%) | 2 (22%) |  |
| Citalopram | 2 (10%) | 0 (0%) | 2 (22%) |  |
| Amitriptyline | 1 (5.0%) | 1 (9.1%) | 0 (0%) |  |
| Citalopram / Mirtazipine | 1 (5.0%) | 1 (9.1%) | 0 (0%) |  |
| Fluoxetine | 1 (5.0%) | 1 (9.1%) | 0 (0%) |  |
| Quietiapine | 1 (5.0%) | 1 (9.1%) | 0 (0%) |  |
| Sertraline | 1 (5.0%) | 1 (9.1%) | 0 (0%) |  |
| Venlafaxine | 1 (5.0%) | 1 (9.1%) | 0 (0%) |  |
| Venlafaxine / Mirtazapine | 1 (5.0%) | 1 (9.1%) | 0 (0%) |  |
| Vortioxetine | 1 (5.0%) | 1 (9.1%) | 0 (0%) |  |
| Participant groups were determined at screening using the Patient Health Questionnaire (PHQ-9) score as High Depression Severity (HD, PHQ-9 ≥ 15 and history of using antidepressant medication) or Low Depression Severity (LD, PHQ-9 ≤ 7). ART: antiretroviral therapy. | | | | |

***Supp Table 2 |*** *Summary statistics for all neuroimaging parameters and blood proteins.*

| **Parameter** | **MRS Referencing** | ***n*** | **median** | **Q1** | **Q3** |
| --- | --- | --- | --- | --- | --- |
| ADC NAA |  | 20 | 0.00010914 | 0.00010326 | 0.00012495 |
| ADC Creatine | | 20 | 0.00012161 | 0.00011775 | 0.00012628 |
| ADC Choline | | 20 | 0.00011288 | 0.00010162 | 0.0001187 |
| K_trans_ Whole Brain | | 19 | 0.000848 | 0.0007995 | 0.0009755 |
| Aspartate | Creatine | 20 | 0.33 | 0.32 | 0.36 |
| Choline | Creatine | 19 | 0.23 | 0.19 | 0.28 |
| Glucose | Creatine | 20 | 1.50 | 1.44 | 1.59 |
| Glutamate + Glutamine | Creatine | 19 | 0.80 | 0.75 | 0.85 |
| Lactate | Creatine | 20 | 0.09 | 0.08 | 0.10 |
| Myo-Inositol | Creatine | 20 | 1.26 | 1.19 | 1.31 |
| NAA | Creatine | 20 | 0.26 | 0.24 | 0.28 |
| Aspartate | Water | 20 | 3.11 | 2.86 | 3.51 |
| Choline | Water | 20 | 4.92 | 4.79 | 5.19 |
| Creatine | Water | 19 | 2.08 | 1.82 | 2.56 |
| Glucose | Water | 20 | 14.15 | 13.74 | 15.23 |
| Glutamate + Glutamine | Water | 19 | 7.60 | 7.38 | 8.05 |
| Lactate | Water | 20 | 0.85 | 0.75 | 0.96 |
| Myo-Inositol | Water | 20 | 11.96 | 11.48 | 12.37 |
| NAA | Water | 20 | 2.59 | 2.30 | 2.75 |
| BDNF |  | 18 | 181.69 | 115.95 | 342.32 |
| CD62E |  | 19 | 22408.01 | 15147.86 | 29376.33 |
| CD62P |  | 20 | 564809.63 | 365576.18 | 973399.40 |
| CRP |  | 16 | 2208.73 | 1402.81 | 5145.28 |
| ICAM1 |  | 18 | 122256.52 | 44345.02 | 227382.31 |
| IFN.alpha |  | 19 | 0.27 | 0.10 | 1.56 |
| IL1.alpha |  | 20 | 0.12 | 0.02 | 0.61 |
| IL1.beta |  | 17 | 0.11 | 0.01 | 2.02 |
| IL12p70 |  | 19 | 60.93 | 50.28 | 84.21 |
| IL17A |  | 16 | 10.57 | 5.55 | 17.36 |
| IL4 |  | 20 | 5.02 | 3.70 | 9.34 |
| IL8 |  | 19 | 0.43 | 0.10 | 1.32 |
| IP10 |  | 19 | 6.82 | 4.77 | 9.47 |
| MCP1 |  | 20 | 74.17 | 51.78 | 115.92 |
| MIG |  | 15 | 3.28 | 2.12 | 4.42 |
| MIP1.alpha |  | 18 | 1.12 | 0.44 | 1.64 |
| MIP1.beta |  | 19 | 50.61 | 39.36 | 64.15 |
| NGF.beta |  | 20 | 0.58 | 0.51 | 0.66 |
| TNF.alpha |  | 19 | 31.94 | 20.14 | 62.57 |
| YKL40 |  | 15 | 60.75 | 53.46 | 748.74 |

ADC: apparent diffusion coefficient.

***Supp Table 3 |*** *Spearman’s correlations for all neuroimaging parameters and depressive symptom severity****,*** *adjusted for years of education or CD4 count****.***

| **Covariate(s)** | **Parameter** | **MRS Referencing** | ***n*** | ***ρ*** | ***p*** | ***p*_FDR_** |
| --- | --- | --- | --- | --- | --- | --- |
| Education + fWM | ADC NAA |  | 20 | 0.02 | 0.945 | 0.945 |
| Education + fWM | ADC Creatine | | 20 | 0.25 | 0.25 | 0.324 |
| Education + fWM | ADC Choline | | 20 | -0.11 | -0.11 | 0.651 |
| Education | K_trans_ Whole Brain | | 19 | -0.08 | 0.752 | 0.752 |
| Education | K_trans_ Thalamus | | 18 | -0.45 | 0.068 | 0.137 |
| Education + fWM | Aspartate | Creatine | 20 | 0.51 | 0.032 | 0.227 |
| Education + fWM | Choline | Creatine | 20 | -0.19 | 0.459 | 0.748 |
| Education + fWM | Glucose | Creatine | 19 | 0.04 | 0.893 | 0.893 |
| Education + fWM | Glutamate + Glutamine | Creatine | 20 | 0.14 | 0.585 | 0.748 |
| Education + fWM | Lactate | Creatine | 20 | 0.12 | 0.642 | 0.748 |
| Education + fWM | Myo-Inositol | Creatine | 19 | -0.34 | 0.178 | 0.625 |
| Education + fWM | NAA | Creatine | 20 | -0.13 | 0.614 | 0.748 |
| Education + fWM | Aspartate | Water | 20 | 0.41 | 0.094 | 0.485 |
| Education + fWM | Choline | Water | 20 | 0.02 | 0.950 | 0.950 |
| Education + fWM | Creatine | Water | 20 | 0.04 | 0.883 | 0.950 |
| Education + fWM | Glucose | Water | 19 | 0.04 | 0.888 | 0.950 |
| Education + fWM | Glutamate + Glutamine | Water | 20 | 0.15 | 0.548 | 0.950 |
| Education + fWM | Lactate | Water | 20 | 0.11 | 0.668 | 0.950 |
| Education + fWM | Myo-Inositol | Water | 19 | -0.39 | 0.121 | 0.485 |
| Education + fWM | NAA | Water | 20 | -0.07 | 0.796 | 0.950 |
| CD4 + fWM | ADC NAA |  | 20 | 0.25 | 0.315 | 0.473 |
| CD4 + fWM | ADC Creatine | | 20 | 0.46 | 0.45 | 0.061 |
| CD4 + fWM | ADC Choline | | 20 | 0.03 | 0.01 | 0.977 |
| CD4 | K_trans_ Whole Brain | | 19 | -0.09 | 0.735 | 0.735 |
| CD4 | K_trans_ Thalamus | | 18 | -0.41 | 0.101 | 0.202 |
| CD4 + fWM | Aspartate | Creatine | 20 | 0.41 | 0.088 | 0.619 |
| CD4 + fWM | Choline | Creatine | 20 | -0.15 | 0.559 | 0.973 |
| CD4 + fWM | Glucose | Creatine | 19 | -0.08 | 0.746 | 0.973 |
| CD4 + fWM | Glutamate + Glutamine | Creatine | 20 | 0.24 | 0.340 | 0.973 |
| CD4 + fWM | Lactate | Creatine | 20 | -0.03 | 0.892 | 0.973 |
| CD4 + fWM | Myo-Inositol | Creatine | 19 | -0.11 | 0.680 | 0.973 |
| CD4 + fWM | NAA | Creatine | 20 | -0.01 | 0.973 | 0.973 |
| CD4 + fWM | Aspartate | Water | 20 | 0.24 | 0.344 | 0.707 |
| CD4 + fWM | Choline | Water | 20 | -0.19 | 0.442 | 0.707 |
| CD4 + fWM | Creatine | Water | 20 | -0.22 | 0.377 | 0.707 |
| CD4 + fWM | Glucose | Water | 19 | -0.08 | 0.751 | 0.858 |
| CD4 + fWM | Glutamate + Glutamine | Water | 20 | -0.02 | 0.932 | 0.932 |
| CD4 + fWM | Lactate | Water | 20 | -0.13 | 0.612 | 0.816 |
| CD4 + fWM | Myo-Inositol | Water | 19 | -0.43 | 0.085 | 0.684 |
| CD4 + fWM | NAA | Water | 20 | -0.32 | 0.198 | 0.707 |

ADC: apparent diffusion coefficient; fWM: fractional white matter volume.

***Supp Table 4 |*** *Spearman’s correlations for all neuroimaging parameters and blood proteins with depressive symptom severity.*

| **Parameter** | **MRS Referencing** | ***ρ*** | ***p*** | ***p*_FDR_** |
| --- | --- | --- | --- | --- |
| Aspartate | Creatine | 0.54 | 0.016 | 0.115 |
| Choline | Creatine | -0.13 | 0.599 | 0.973 |
| Glucose | Creatine | 0.00 | 0.987 | 0.987 |
| Glutamate + Glutamine | Creatine | 0.15 | 0.549 | 0.973 |
| Lactate | Creatine | 0.15 | 0.541 | 0.973 |
| Myo-Inositol | Creatine | -0.10 | 0.695 | 0.973 |
| NAA | Creatine | -0.01 | 0.975 | 0.987 |
| Aspartate | Water | 0.43 | 0.066 | 0.527 |
| Choline | Water | -0.04 | 0.858 | 0.970 |
| Creatine | Water | -0.12 | 0.638 | 0.970 |
| Glucose | Water | 0.01 | 0.970 | 0.970 |
| Glutamate + Glutamine | Water | 0.03 | 0.918 | 0.970 |
| Lactate | Water | 0.13 | 0.587 | 0.970 |
| Myo-Inositol | Water | -0.29 | 0.243 | 0.970 |
| NAA | Water | -0.08 | 0.741 | 0.970 |
| K_trans_ Whole Brain | | -0.15 | 0.537 | 0.537 |
| K_trans_ Thalamus | | -0.37 | 0.129 | 0.257 |
| ADC Choline | | 0.24 | 0.312 | 0.467 |
| ADC Creatine | | 0.48 | 0.034 | 0.102 |
| ADC NAA |  | 0.07 | 0.768 | 0.768 |
| BDNF |  | -0.09 | 0.719 | 0.899 |
| CD62E |  | 0.1 | 0.674 | 0.899 |
| CD62P |  | 0.29 | 0.211 | 0.824 |
| CRP |  | 0.25 | 0.345 | 0.824 |
| ICAM1 |  | 0.22 | 0.379 | 0.824 |
| IFN.alpha |  | 0.02 | 0.927 | 1 |
| IL1.alpha |  | 0.14 | 0.567 | 0.899 |
| IL1.beta |  | 0.21 | 0.412 | 0.824 |
| IL12p70 |  | 0.25 | 0.293 | 0.824 |
| IL17A |  | 0 | 1 | 1 |
| IL4 |  | 0.1 | 0.662 | 0.899 |
| IL8 |  | 0.02 | 0.942 | 1 |
| IP10 |  | -0.24 | 0.314 | 0.824 |
| MCP1 |  | 0.01 | 0.955 | 1 |
| MIG |  | 0.11 | 0.707 | 0.899 |
| MIP1.alpha |  | -0.17 | 0.501 | 0.899 |
| MIP1.beta |  | 0.23 | 0.354 | 0.824 |
| NGF.beta |  | 0.48 | 0.032 | 0.64 |
| TNF.alpha |  | 0.29 | 0.228 | 0.824 |
| YKL40 |  | -0.28 | 0.306 | 0.824 |

***Supp Table 5*** *| Cramer-Rao Lower Bounds (CLRB) values for MRS data.*

| **Metabolite** | **CRLB mean (SD)** |
| --- | --- |
| Asp | 10.9 (2.7) |
| Cr | 7.0 (8.0) |
| Glc | 9.3 (3.0) |
| Gln | 9.2 (1.9) |
| Glu | 2.5 (0.6) |
| GPC | 2.2 (0.4) |
| Ins | 3.4 (0.8) |
| Lac | 19.8 (6.6) |
| PCr | 7.0 (6.2) |
| PCho | 999.0 (0) |
| NAA | 1.4 (0.5) |
| NAAG | 164.5 (305.8) |
| NAA+NAAG | 1.1 (0.2) |
| Cr+PCr | 1.1 (0.3) |
| Glu+Gln | 2.4 (0.6) |
| PCho+GPC | 2.2 (0.4) |

***Supp Table 6*** *| Cramer-Rao Lower Bounds (CLRB) for DW-MRS data.*

|  | **CRLB mean (SD)** | | |
| --- | --- | --- | --- |
| **b (s/mm2)** | *tNAA* | *tCr* | *tCho* |
| 0 | 2.2 (0.4) | 2.5 (0.5) | 3.7 (1.0) |
| 1863 | 2.1 (0.3) | 2.4 (0.5) | 3.7 (1.0) |
| 3856.5 | 2.3 (0.5) | 2.9 (0.5) | 4.4 (1.9) |
